# Towards a Machine Learning-Based Digital Twin for Non-Invasive Human Bio-Signal Fusion

**DOI:** 10.3390/s22249747

**Published:** 2022-12-12

**Authors:** Izaldein Al-Zyoud, Fedwa Laamarti, Xiaocong Ma, Diana Tobón, Abdulmotaleb El Saddik

**Affiliations:** 1 Multimedia Communications Research Laboratory (MCRLab), School of Electrical Engineering and Computer Science, University of Ottawa, Ottawa, ON K1N 6N5, Canada; 2 Mohamed bin Zayed University of AI, Abu Dhabi, United Arab Emirates; 3 Faculty of Engineering, University of Medellín, Medellín 050010, Colombia

**Keywords:** bio-signal fusion, computer vision, digital health, digital twin, machine learning, metaverse

## Abstract

Human bio-signal fusion is considered a critical technological solution that needs to be advanced to enable modern and secure digital health and well-being applications in the metaverse. To support such efforts, we propose a new data-driven digital twin (DT) system to fuse three human physiological bio-signals: heart rate (HR), breathing rate (BR), and blood oxygen saturation level (SpO2). To accomplish this goal, we design a computer vision technology based on the non-invasive photoplethysmography (PPG) technique to extract raw time-series bio-signal data from facial video frames. Then, we implement machine learning (ML) technology to model and measure the bio-signals. We accurately demonstrate the digital twin capability in the modelling and measuring of three human bio-signals, HR, BR, and SpO2, and achieve strong performance compared to the ground-truth values. This research sets the foundation and the path forward for realizing a holistic human health and well-being DT model for real-world medical applications.

## 1. Introduction

A Digital Twin (DT) is the virtual representation of a living entity (e.g., human beings, animals, plants, etc.) or nonliving entity (e.g., business model, product, process, system, event, machine, building, etc.) that allows real-time interaction and communication between both the real twin and the digital twin to help with the modelling, monitoring, understanding, and optimization of the functions and behaviour of the real twin [1].

The development of high-fidelity virtual models (i.e., digital twins) is a critical step so that the behaviours and characteristics of the real twin can be simulated. DT can be developed using data-driven approaches, mathematical modelling approaches, or hybrid approaches (data and mathematical modelling).

DT technology, which we consider one of the primary technologies required to build and advance the metaverse, has already been used extensively in the manufacturing industry, but it is still in its infancy in the domain of human health and well-being [2,3].

For example, in the industrial manufacturing context, a state-of-the-art open-source framework and software tools for designing DTs (the IoTwins platform) have been proposed to enable users to develop, configure, and run DTs using distributed DT infrastructures based on the IoT, edge computing, and industrial cloud technologies [4]. The DT IoTwins architecture is composed of three main computing infrastructure interactive layers; the IoT layer, the edge layer and the cloud layer. The IoT layer assists the DT with configuring and conducting computing/processing activities on the IoT device’s sensory data. The edge layer is responsible for data processing configuration, bulk data processing, data stream processing, and machine learning model execution. The cloud layer can perform the same functions as the edge layer, but the user can configure and run computing operations on data streams from the IoT or edge devices, as well as data stored in the cloud [4].

Two recent surveys reviewed recent research with an emphasis on building DTs within the context of Industry 4.0. The first survey focused on covering and summarizing the methodological approaches used for designing, modelling, and implementing DTs, including details on how to synchronize real-time data between a DT’s physical and virtual components, as well as the current progress of providing DT implementation tools and an experimental testbed [5]. The second survey presented a generic DT application architecture consisting of five layers to describe and specify the basic design requirements, related technologies, and integration interlayer process, with an emphasis on how to upgrade the legacy system in Industry 4.0 technologies to improve productivity, reduce costs, and upgrade the industrial process devices [6].

Human DT is a new research area and has started attracting more attention recently. The human DT concept is mainly based on the collection of real twin (human) data and modelling it using machine learning (ML), a sub-field of Artificial Intelligence (AI), with the goal of extracting information pertaining to the real twin (human)’s health and well-being [1,7,8]. This data collection in the domain of human health generally translates into a challenge for the individual to use and/or wear cumbersome health-sensing devices, which may cause discomfort to individuals and create an additional risk of disease transition.

Understating the concept of the digital twin and how it is implemented in the human DT domain is a challenging new research area, and there is a clear gap in the demonstration of how to design and implement DT technology for human health and well-being applications at the granular level.

Bio-signals, such as heart rate (HR), breathing rate (BR), blood oxygen saturation level (SpO2), and blood pressure (BP), can be used to diagnose human physical and psychological health states such as emotions, fatigue, heart rate variability, mental stress, and sleep patterns [9]. These bio-signals are becoming crucial to accelerating the adaptation of virtual health and telemedicine services in the healthcare system, which have gained huge momentum in the post-pandemic era.

The authors of another more recent comprehensive survey [10] revealed several gaps in the area of the remote measurement of heart rate using machine learning methods outside the context and area of human DT. These gaps are that (a) the literature focuses on a single vital sign and there is a need to fuse other bio-signals such as SpO2, (b) the proposed solutions are complex and cannot be applied in a real-time practical application, and (c) there is a need for diverse datasets that contain skin-tone variations.

To address all above mentioned gaps and to establish the groundwork and path forward for achieving a holistic human health and well-being DT model for practical virtual healthcare services in the metaverse, we propose building a data-driven digital twin solution capable of sensing and fusing human bio-signals remotely using modern computer vision/image processing and remote photoplethysmography (rPPG) technologies.

rPPG is considered a low-cost and accessible optical sensing technique that can detect tiny colour intensity changes caused by changes in facial blood flow due to cardiovascular activities under an ambient light environment. Such changes are invisible to the human eye but can be captured directly by a regular colour digital camera sensor. By capturing video and analyzing the data in red, green, and blue (RGB) channels, multiple bio-signals can be extracted from a distance [11].

Our proposed DT technology has the potential to be applied to real-world applications, e.g., (a) in the emergency room in a hospital for quick bio-signal pre-screening processes, and (b) in long-term health units to monitor and detect elderly patients’ health and well-being signs remotely, and act as a diagnostic support tool that can raise the alarm as soon as an anomaly (i.e., abnormal health pattern) is detected to prevent further chronic problems and degradation in patients’ health.

The structure of this paper is as follows. Section 2 describes the methods and materials used, and Section 3 highlights our proposed DT system design and the developed framework utilized to perform offline ML experimentation, development, and testing on ML models required for multi-bio-signal modelling. Section 4 presents the achieved results and the discussion presented in Section 5, along with limitations and future work. Finally, Section 6 presents the conclusion.

## 2. Materials and Methods

This section describes the dataset engineering; applied machine learning algorithms; proposed DT system architecture including a framework for ML offline experimentation, development, and testing; and evaluation metrics used to demonstrate the performance of our proposed DT system.

### 2.1. Datasets

We used three biometric datasets, two from the public domain (VIPL and COHFACE) and one from our lab as detailed below.

#### 2.1.1. VIPL Public Dataset

The VIPL dataset was developed by the Key Laboratory of Intelligent Information Processing of the Chinese Academy of Sciences [12] and is considered a large-scale multi-modal dataset that contains 2378 visible-light video clips and 752 near-infrared video clips of 107 participants (subjects), with a female-to-male gender ratio of about 30% to 70%. Only the visible-light videos and their corresponding heart-rate and SpO2 biological data are used in this research. Unfortunately, this dataset does not contain any BR data.

#### 2.1.2. COHFACE Public Dataset

Portions of the research in this paper used the COHFACE dataset. This dataset was developed by IDIAP Research Institute in Switzerland and contains 160 one-minute-long RGB video sequences of 40 participants, including 12 females and 28 males. The dataset includes synchronized data on blood volume pulse (BVP) and BR. However, by now, the original recorded video files have become highly compressed and noisy.

#### 2.1.3. Our Lab Dataset

We collected and engineered our lab dataset, which includes 197 video clips for 18 participants varying in gender, race, and age with synchronized heart-rate and breathing-rate biological data. The video clips were collected using a regular colour camcorder at a frame rate of 30 FPS (frames per second). Compared to the other datasets used, it is noteworthy to mention that our physical data collection contains an extended heart-rate range. The recordings of the subjects were made in both sitting-still and exercising states [13].

The purpose of adding our dataset is to enrich the training, validation, and testing datasets with heart-rate and breathing-rate (which was not available in the previous two public datasets) data and to address the gaps in the most recent literature survey [10] regarding the need for a diverse dataset with more skin-tone variations.

### 2.2. ML Algorithms

In this research, the bio-signal measurement technique is formulated as an ML regression modelling problem and we address it by investigating the following three advanced supervised ML algorithms using the Python TensorFlow open-source library.

#### 2.2.1. Multilayer Perceptron Algorithm

The multilayer perceptron (MLP) neural network is a supervised deep learning algorithm that can learn a nonlinear function that maps input features to the target outputs. Classical MLP networks consist of an input layer, a hidden layer(s), and an output layer.

In our research, we extended the classical MLP and utilized additional layers such as batch normalization and regularization layers. The objective behind this was to design a robust learning-based DT system capable of performing an optimal real-time fusion of multiple bio-signals from a single input feature space.

From an implementation perspective, our MLP neural network consists of the following fully connected sequential layers [14]:

1. The input (dense) layer is designed to consume all the input features for each bio-signal, as defined by our problem in the upcoming sections.
2. The batch normalization layer is fitted right after the input layer and performs a scaling/normalizing of the raw input features instead of manual data preprocessing. This layer provides us with two major advantages: (i) the achievement of faster training convergence because it enables the network to use higher learning rates and reduces the effect of initialization, and (ii) the ability to use regularization services as a bonus through a reduction in generalization errors, i.e., improving network performance generalization to unseen data [15].
3. The activation layer refers to adding a nonlinear activation function to the outputs of the batch normalization layer. The selection of the activation function is crucial since it helps the network to learn the complex nonlinear relationship between the input and output features. It improves the capability of MLP generalization during testing/operations on new data. We employed a simple, well-known, computationally efficient activation function called a rectified linear unit (ReLU) in all dense layers, except for the output layer. Proper network weight initialization is a very important step to prevent activation function outputs from vanishing or exploding during training. We applied weight initialization in the input, hidden, and output layers.
4. The regularization layer is implemented using the dropout technique. Regularization helps the neural network to learn from training data and generalize well on the test data rather than memorize the training data and fail to generalize on the test data (which is called overfitting). Dropout improves the network training performance and minimizes overfitting by removing (dropping out) the contributions of a random number of neurons in the next layer by a certain dropout rate. Dropout can be used after an input or hidden layer but not after an output layer [16].
5. Dense (hidden) layers with different sizes.
6. One output layer with one neuron and the linear activation function to measure the actual value of each bio-signal since we are implementing a regression solution in this research. We train the above MLP neural network using a state-of-the-art stochastic gradient-based optimization algorithm called “Adam”. The advantages of this algorithm are that it provides (i) computational efficiency through significant fast convergence compared to standard gradient-based descent algorithms, (ii) efficient memory requirements, and (iii) scalability to a large dataset and large ML models [17]. These advantages enable the MLP to perform online (real-time) learning capabilities and are what motivated us to consider MLP in our research since one of the main DT system requirements is the ability to have real-time interaction/communication with the real twin.

#### 2.2.2. Long Short-Term Memory Algorithm

The long short-term memory (LSTM) algorithm is a special type of recurrent neural network (RNN). RNNs cannot store information for a long time or learn long-term temporal dependencies between inputs and outputs when delays exist. RNNs’ short-term memory creates two major challenges for the recurrent backpropagation training algorithm’s gradient: vanishing and exploding gradients. A memory unit was introduced to address these RNN challenges. The memory unit helped to memorize inputs for a long time and learn the long “short-term” dependency between the input and the output [18]. Such an architecture enabled this special RNN to demonstrate outstanding performance in solving sequence modelling problems in audio and video analytic domains, which motivated us to investigate such a network.

Our research extended the classical LSTM network and utilized additional layers: a batch normalization layer, MLP dense layers, and regularization layers. From an implementation perspective, the following sequential architecture was considered:

1. The LSTM layer. The function of this layer is to perform point-wise multiplication/addition operations to smooth the gradient flow over long sequences and learn the long-term time-series and sequence data dependencies during training. This layer consists of shaped input features, the dimensionality of the output space, activation, initialization, and internal dropout attributes.
2. The batch normalization layer. This layer comes right after the LSTM layer. It performs the scaling/normalizing of the output of the LSTM layer, which has the same advantages as those stated earlier in the MLP section.
3. Multiple MLP cascaded down dense layers. The dense layers help us to further integrate the output from the batch normalization/LSTM layer. A leaky rectified linear unit (LeakyReLU) was added after each dense layer as an activation function. We applied weight initialization in the input, hidden, and output layers.
4. The regularization layer. This layer is implemented using the dropout technique and has the same advantages as those stated earlier in the MLP section.
5. The output layer. One output layer with one unit is equipped with a linear activation function to measure the actual value of each bio-signal since we are implementing a regression solution in this research.

#### 2.2.3. Extreme Gradient Boosting Algorithm

The extreme gradient-boosting algorithm (XGBoost) is based on the traditional ensemble technique. When solving the regression problem, additive models (i.e., decision trees) are sequentially added until the minimized loss by the gradient descent algorithm stops improving. XGBoost was developed originally by Chen Tianqi [19]. The simplicity of its implementation and its exceptional performance in speed and accuracy, especially in winning ML competitions, have motivated us to use it in our research. We utilized the simple algorithm of XGBoost.

### 2.3. Evaluation Metrics

We implemented four performance evaluation metrics in our experiments to evaluate the training, validation, and testing performance of the developed ML models. These metrics are:

(1) $$\mathrm{Mean}\;\mathrm{absolute}\;\mathrm{error}\;\left(\mathrm{MAE}\right)=\frac{1}{n}(\sum_{i=0}^{n}|y_{i}^{g}-y_{i}^{p}\left|\right)$$

(2) $$\mathrm{Mean}\;\mathrm{absolute}\;\mathrm{percentage}\;\mathrm{error}\;\left(\mathrm{MAPE}\right)=\frac{1}{n}\left(\sum_{i=0}^{n}\frac{|y_{i}^{g}-y_{i}^{p}|}{y_{i}^{g}}\right)\times 100$$

(3) $$\mathrm{Root}\;\mathrm{mean}\;\mathrm{square}\;\mathrm{error}\;\left(\mathrm{RMSE}\right)=\sqrt{\left(\sum_{i=0}^{n}\frac{{\left(y_{i}^{g}-y_{i}^{p}\right)}^{2}}{n}\right)}$$

(4) $$\mathrm{Pearson}\;\mathrm{correlation}\;\mathrm{coefficient}=\frac{\frac{1}{\left(n-1\right)}\left(\sum_{i=0}^{n}\left(\left(y_{i}^{g}-mean\left(y_{i}^{g}\right)\right)\times \left(y_{i}^{p}-mean\left(y_{i}^{p}\right)\right)\right)\right)}{\mathrm{STD}\left(y_{i}^{g}\right)\times \mathrm{STD}\left(y_{i}^{p}\right)}$$

where $y_{i}^{g}$ is the ground truth bio-signal values, $y_{i}^{p}$ is the predicted (measured) bio-signal values, *n* is the number of data points, and STD is the standard deviation.

## 3. Digital Twin System Design

This work’s scientific contribution focuses on leveraging data-driven digital twin technology and how to implement it in the human digital twin domain for health and well-being applications, which is a very new area in the literature at this granular level.

**Figure 1:**
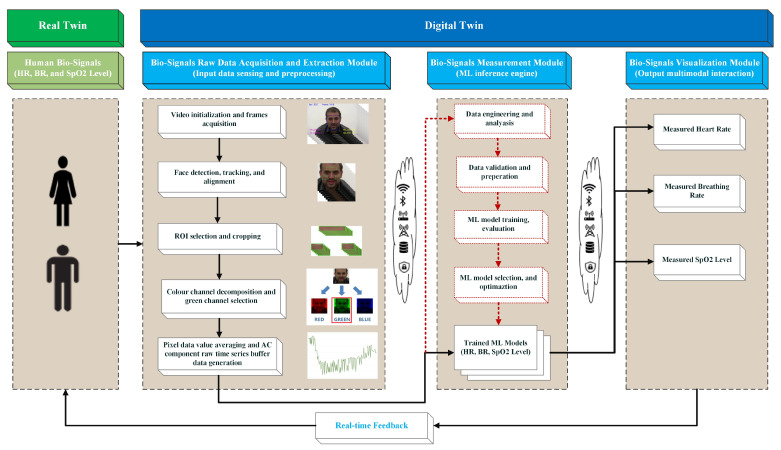
System Architecture of the Machine Learning-based Digital Twin for Non-Invasive Human Bio-Signal Fusion.

We designed our DT system using a simple architecture that consists of three integrated modules, as illustrated in Figure 1. The bio-signal data extraction module is responsible for converting raw video frames into time-series data.

The bio-signal measurement module is responsible for processing the raw time-series data and producing each bio-signal using optimized ML-trained models. The bio-signal visualization and interaction module represents the last part of the DT system and should have the ability to provide real-time updates to all three bio-signal readings on a device screen.

The communication between the DT system’s modules is executed using Bluetooth, Wi-Fi/5G networks, or conventional communications services. We implemented the integrated modules using a Python-integrated development environment (PyCharm). The upcoming sub-sections provide an in-depth look at the processes of the mechanics of each module.

### 3.1. Bio-Signal Raw Data Acquisition and Extraction Module

This module is responsible for the sensing, preprocessing, and extraction of bio-signal raw data from video images taken either directly from a digital camera (real-time mode) or pre-recorded (offline mode) and generates a single time-series output (e.g., 150 green-channel AC-component data points). The inputs of this module are raw video frames (e.g., 150 frames) acquired from a set of short video clips, with synchronized bio-signal ground-truth data taken from each dataset (e.g., a total of 2378 video clips from the VIPL dataset).

The outputs are time-series data per region of interest (ROI) per image (e.g., 150 green-channel AC-component data points) stored in a set of files in “csv” format with essential formatted information (e.g., a total of 7134 files for all three ROIs were generated from the VIPL dataset). Each file contains the participant numbers and physical ground truth for the applicable bio-signal, and 150 points of buffered data are extracted and synchronized with each physical ground truth. The steps we executed during this stage are discussed below.

#### 3.1.1. Video Source (Camera) Initialization and Video Frame Acquisition

There are two types of inputs that can be used as video sources: (i) image frames collected in real-time from a computer webcam, or (ii) image frames collected from pre-recorded offline video files. The system takes one frame from the source until the video-capturing or file-reading process ends.

#### 3.1.2. Face Detection and Tracking

The OpenCV Haar cascade classifier, an open-source Python library, well-known for its effectiveness in real-time object detection applications in computer vision, is applied to detect a human face in the currently processed frame. If an OpenCV facial detector identifies the face on the input video frame, a face frame is tracked and cropped. If the detected face is not correctly positioned in the face frame, we apply an open-source Python facial alignment library (Imutils) to straighten up the face pose before the cropping (i.e., minimize the impact of the head/face pose on the accuracy of dlib for landmark placement). The tracking algorithm resets when the designed buffer is filled or the face is no longer detectable. The motivation behind using the OpenCV Haar cascade classifier in our DT system design research is driven by its proven capability to provide real-time face detection and its availability as a highly optimized open-source Python library.

#### 3.1.3. Facial Landmark Detection and System Authorization

**Figure 2:**
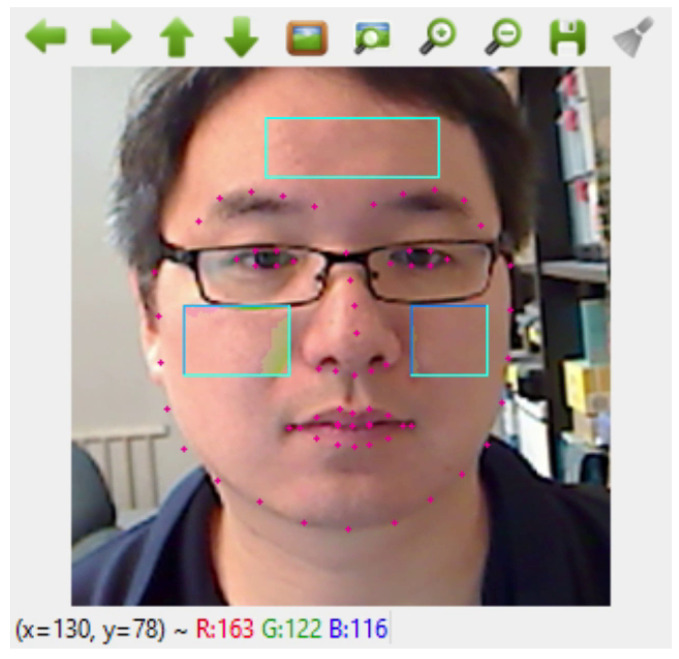
Captured ROIs using Dlib 68-point facial landmarks.

In this stage, we employed a 68-point facial landmark detection open-source Python library (DLib). The DLib pre-trained model uses the regression trees technique to measure 68 facial landmarks based on pixel intensities instead of facial features, producing high-quality face detection in real time (See Figure 2). After the video frame acquisition and face detection, the locations of the facial landmarks are identified and extracted.

#### 3.1.4. Region of Interest (ROI) Cropping and Selection

The DLib 68-point version was used to identify three ROIs on the face frame, such as two on the left and right cheeks and one on the forehead; this is because the signals related to the absorption bands for oxy- and de-oxy hemoglobin had the strongest signal-to-noise ratio (SNR) corresponding to blood volume changes in these facial skin regions [20].

#### 3.1.5. Colour Channel Decomposition and Green-Channel Selection

Previous research [21] indicates that the green-channel data in the RGB colour space remain the most robust for bio-signal extraction (e.g., heart-rate signal extraction). Hence, the green channel is selected as our choice for implementation in the scope of our research due to the presence of the highest signal-to-noise ratio on bio-signal data in this channel.

#### 3.1.6. Pixel Value Averaging and Buffer Data Generation

This step transforms the large sizes of 2D image sequences into a 1D time-series data array and has the advantage of reducing computational expense, despite the reduced size of the input data. The average colour-intensity values based on the green channel and its corresponding ROI images are calculated and stored temporarily in the data buffers.

Each buffer is designed to accept a maximum of 150 data values so it is full when 150 ROI image frames have been successfully received and processed. This takes five seconds at a frame rate of 30 FPS with 0% overlapping between each pair of adjacent windows. The processing time is calculated by dividing the buffer size by the camera frame rate. A frame rate of 30 FPS is the most common frame rate found in many commercial-grade webcams. The sample video clips in all datasets that we utilized in this research were also collected at a frame rate of 30 FPS.

The buffer length was designed after performing a massive number of experiments to achieve optimal performance of the DT real-time system by providing enough data samples and retaining accuracy while updating the bio-signal measurements in nearly real time. By choosing 150-point data as the size of the buffer window, the DT system could run and update bio-signal readings efficiently in real time and retain at least one cycle of the BR and usually five cycles of the HR. This is a crucial factor for the design of an accurate system running in real time, even with hardware constraints.

In addition, from a signal-processing perspective, the sampling rate needs to be fast enough to enable the detection of the heart rate at its maximum possible frequency, which we have set at 180 BPM or 3Hz. According to the Nyquist limit, the highest frequency component that can be accurately reconstructed should be equal to or less than half of the sampling rate [22].

A frame rate of 30 FPS is not a mandatory camera speed since our system sampling rate is determined by how fast our data buffer is filled during the digital camera video recording in real time. Therefore, a minimum of 6 FPS (or 6 Hz) as a frame acquisition speed should be maintained to perform accurate bio-signal measurements for obtaining an HR at a detection mode of 180 BPM or less.

### 3.2. Bio-Signal Data Measurement Module

This module represents the ML inference engine, which we consider the core of the DT, and has two main functions: (i) perform data and ML model engineering in offline experimentation setting to produce optimized, trained ML models, as indicated in Figure 1 by the red dotted communication lines and boxes, and (ii) consume the raw time-series-shaped buffer data (e.g., 150 green-channel AC-component data points) and produce three bio-signal measurements simultaneously in real time using the optimized trained ML models. Each step of the process is explained in more depth below.

#### 3.2.1. Data Engineering and Analysis

The input of this stage is a set of raw time-series data files in “csv” format and the outputs are three engineered raw datasets (i.e., VIPL, COHFACE, and our lab dataset). The steps of the data engineering and analysis stage are (i) aggregating all the generated raw time-series data (green-channel AC-component data) from the previous stage for each dataset; before data aggregation, empty files that resulted from no face detection are sorted and filtered; (ii) performing exploratory data analysis (EDA) to analyze and understand the statistical characteristics of each bio-signal dataset and identify potential outliers before model experimentation; and (iii) producing three engineered datasets: the VIPL dataset that contains HR and SpO2 bio-signal data, our lab dataset that contains HR and BR bio-signal data, and COHFACE that contains HR and BR bio-signal data.

#### 3.2.2. Data Validation and Preparation

The inputs of this stage are three datasets (i.e., VIPL, COHFACE, and our lab datasets) and the outputs are three clean (training, validation, and testing) datasets for each bio-signal (i.e., HR, BR, and SpO2). The steps of the data preparation stage are (i) performing dataset cleaning based on the observations highlighted in the previous stage, as well as ensuring all three datasets are free of rows with zero values if any; (ii) splitting each vital dataset into training (60%), validation (20%), and testing (20%); the data split should include all the subject data in the respected dataset to avoid data leakage (i.e., a case where the participant data are split among the training, validation, and testing datasets). Data leak prevention is essential for producing an accurate model parameter capable of providing predictive power once we deploy them in the production environment; and (iii) aggregating and creating three separate datasets for training, validation, and testing purposes (e.g., the final training dataset is an aggregation of 60% of VIPL dataset, 60% of our lab dataset, and 60% of COHFACE dataset)

The data split was performed by applying filters in Panda’s Python package to maintain proper dataset partitioning, where each participant’s data are present in one dataset and do not appear in other datasets to avoid the leakage of any single participant in the training, validation, and testing datasets. The 60/20/20 division of the data samples generated from all subjects was chosen since the length of each participant video was variable and the generated data samples were not equal among all participants. The percentage refers to the split in the data samples across all samples for each dataset.

#### 3.2.3. ML Model Training and Validation

The input of this stage is a clean training dataset ready to perform experimentation on three ML algorithms (i.e., XGBoost, MLP, and LSTM) and the outputs are three trained models prepared for testing.

The steps of the ML model training and validation stage are (i) establishing a list of potential ML regression algorithms for designing and developing various ML models capable of accurately detecting and estimating bio-signals; (ii) establishing model performance evaluation metrics to assess model prediction quality; (iii) performing model training experimentation based on the engineered training dataset from the previous stage; (iv) performing model validation experimentation based on the engineered validation (holdout) dataset from the previous stage; (v) visualizing the learning profile during training and validation for each ML model per bio-signal; and (vi) performing hyperparameter tuning and producing the best-trained ML models for the upcoming testing stage.

#### 3.2.4. ML Model Testing, Selection, and Tuning

The input of this stage is nine trained ML models with three models per bio-signal (i.e., MLP, LSTM, and XGBoost) and the outputs are the best-three performing models selected for each bio-signal, saved in tuple format and ready for deployment in the operating environment.

The steps of the ML model testing, selection, and tuning stage are (i) establishing performance validation and selection metrics; (ii) analyzing and reporting each model’s testing performance; and (iii) selecting, saving, and transferring the best-performing model’s parameters/weights in tuple format for the system design, deployment, and operation phases.

### 3.3. Bio-Signal Visualization Module

This module represents the last part of the DT twin system and facilitates the output’s visualization and interaction with the real twin through Bluetooth, Wi-Fi/5G networks, or conventional communications services. In our proposed system, the ML-based bio-signal DT model’s output interface should have the capability to provide real-time updates to all three bio-signal readings on a device screen.

## 4. Results

### 4.1. Dataset Engineering and Analysis

The EDA was achieved by visualizing the extracted green-channel AC-component data on each bio-signal using both histograms and box plots and producing a statistical data profile. The figures and tables refer to the original datasets before data cleaning. The entire data engineering and analysis process for each bio-signal was executed using the following sequential steps:

#### 4.1.1. HR Dataset

**Figure 3:**
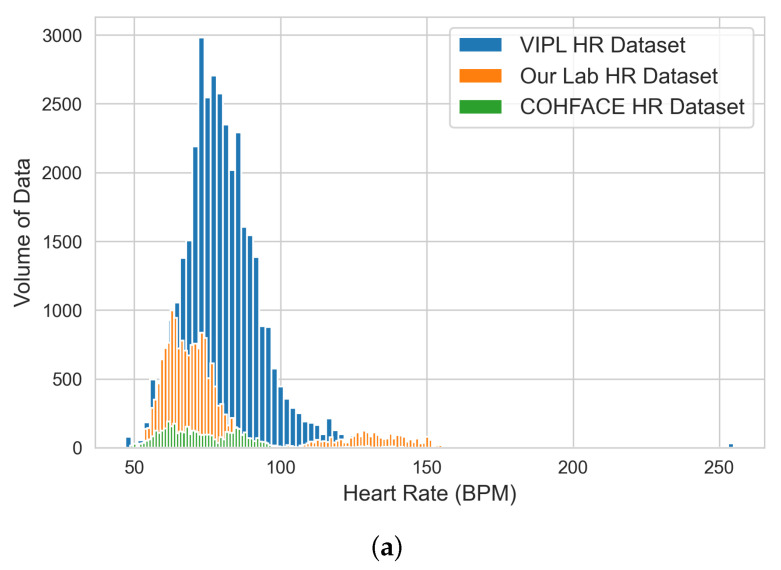

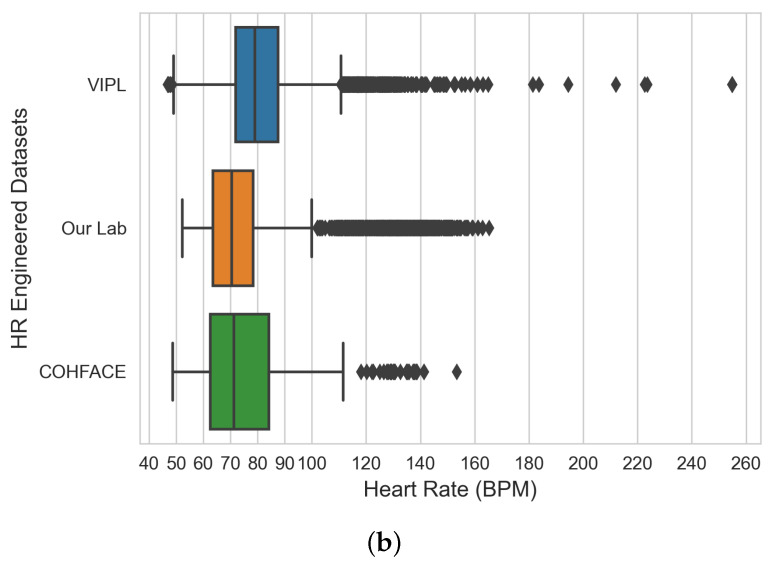
Histogram (**a**) and box plot (**b**) of HR engineered datasets from the extracted green-channel AC-component data.

Our analysis based on the HR data shown in Table 1 and the HR histogram and box plot shown in Figure 3 indicates that (i) the majority of the aggregated physical HR data ranged between 62 and 87 beats per minute (BPM), which means that the trained ML models should perform better within this range. This is in line with the average heart rate for a healthy adult (ranges from 60 to 100 beats per minute). Actual heart rates in real-time applications, however, may increase with exercise, illness, injury, and emotions [23]. Thus, there is likely to be a higher error margin for our models when estimating bio-signals beyond this range, which also dictated the testing criteria for our developed HR ML models in the next stage; (ii) the COHFACE dataset provided the fewest contributions to the overall HR dataset; and (iii) any HR data value greater than 180 BPM was considered an outlier and was cleaned in the next stage before performing ML model training.

#### 4.1.2. BR Dataset

**Figure 4:**
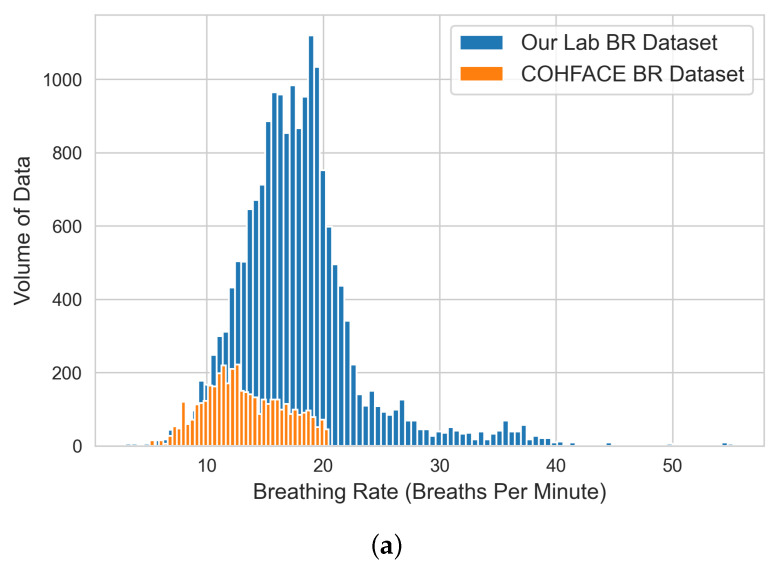

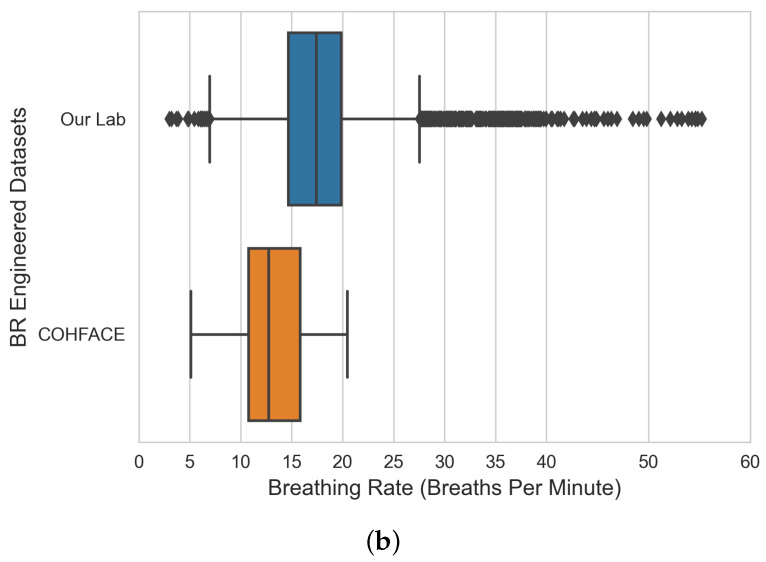
Histogram (**a**) and box plot (**b**) of BR engineered datasets from the extracted green-channel AC-component data.

Our analysis based on the BR data shown in Table 2 and the BR histogram and box plot shown in Figure 4 indicates that (i) the majority of the aggregated physical BR data ranged between 11 and 20 breaths per minute (BPM), which indicates that the trained ML models should perform better within this range (the normal breathing rate for healthy adults ranges from 12 to 16 breaths per minute). The BR can increase with fever, illness, and other medical conditions, thus becoming an important indicator of breathing difficulties [23]). There is likely to be a higher error margin for our models when estimating bio-signals beyond this range, which also dictated the testing criteria for our developed BR ML models in the next stage; (ii) the COHFACE dataset provided the fewest contributions to the overall HR dataset; (iii) any BR data with values greater than 27 BPM and less than 10 BPM were considered anomalies to be cleaned before performing ML model training; and (iv) the VIPL dataset did not contain any BR physical data and thus was excluded from this analysis.

#### 4.1.3. SpO2 Dataset

**Figure 5:**
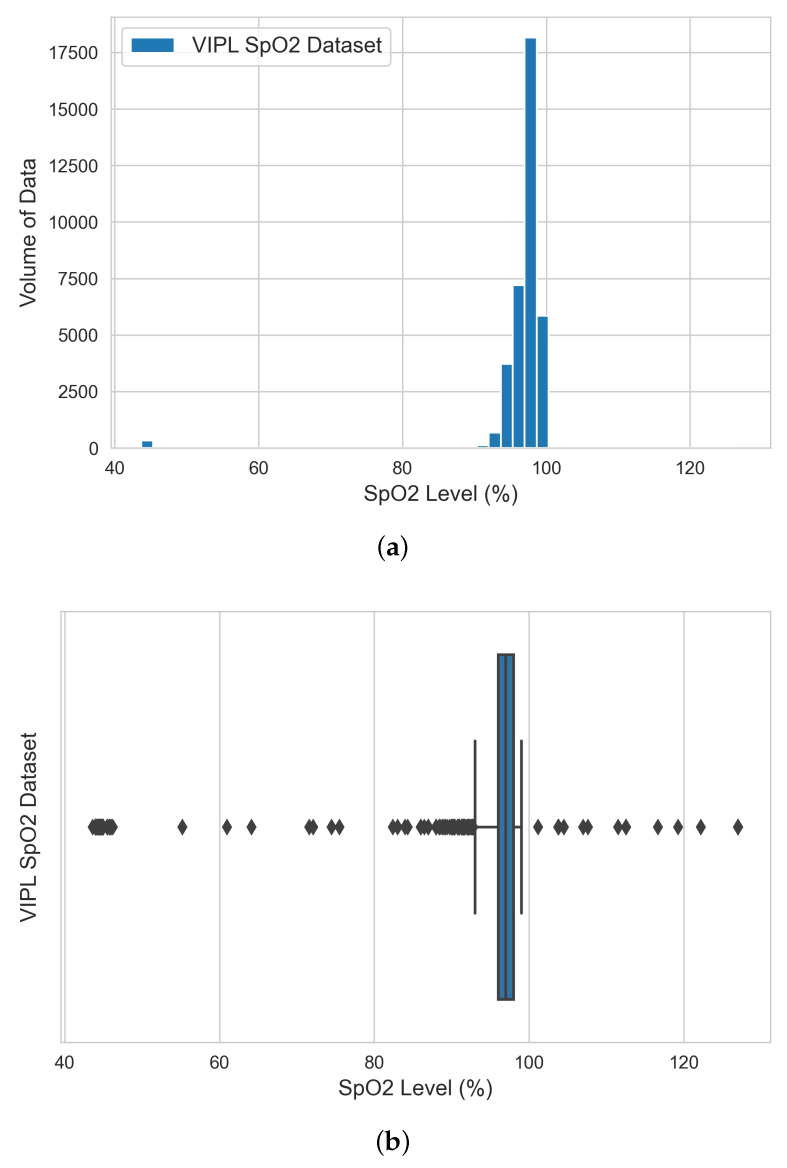
Histogram (**a**) and box plot (**b**) of SpO2 engineered datasets from the extracted green-channel AC-component data.

Our analysis based on the SpO2 data shown in Table 3 and the SpO2 histogram and box plot shown in Figure 5 indicates that (i) only the VIPL dataset contained SpO2 physical data and the remaining datasets were excluded from this analysis; (ii) the majority of the aggregated physical SpO2 data ranged between 96% and 97%, which indicates that the trained ML models should perform better within this range (the normal SpO2 level for healthy adults ranges from 95% to 100%). Low oxygen levels can be an early warning sign that requires medical intervention [23]. There is likely to be a higher error margin for our models when estimating bio-signals beyond this range, which also dictated the testing criteria for our developed SpO2 ML models in the next stage; (iii) any SpO2 data with values greater than 100% and less than 90% were considered anomalies to be cleaned before performing ML model training.

### 4.2. Training and Validation Results

This research was carried out based on three datasets, i.e., our ML model training, validation, and testing were conducted using data from all three datasets based on the proper allocation per bio-signal and an accurate split. The data engineering stage showed that our dataset (18 participants and 197 videos; HR and BR data) produced 18,425 signals/samples and each signal/sample contained 150 data points, which was half of the data generated from the VIPL dataset (36,291 signals/samples, 107 participants and 2378 video clips, HR and SpO2 data) and almost 4 times the volume of data generated from the COHFACE dataset (40 participants and 160 videos, no HR or BR data).

In this stage, we produced two types of learning curves per ML model on each bio-signal: (i) a training learning curve to visualize the model’s learning experience; and (ii) a validating learning curve to visualize the model’s generalizing experience. Such learning curves help us to assess the quality of (a) each model’s learning and generalizing capabilities; and (b) the relative statistical characteristics of the training and validation datasets (diagnosing the problem of unrepresentative datasets).

We used the previously explained four metrics (MAE, MAPE, RMSE, and Pearson correlation coefficient) to evaluate the training and validation performance of the developed ML models. We implemented the “early stopping” technique to stop the model training when the validation loss performance metric stopped improving to prevent overfitting.

#### 4.2.1. HR ML Model Training and Validation

**Figure 6:**
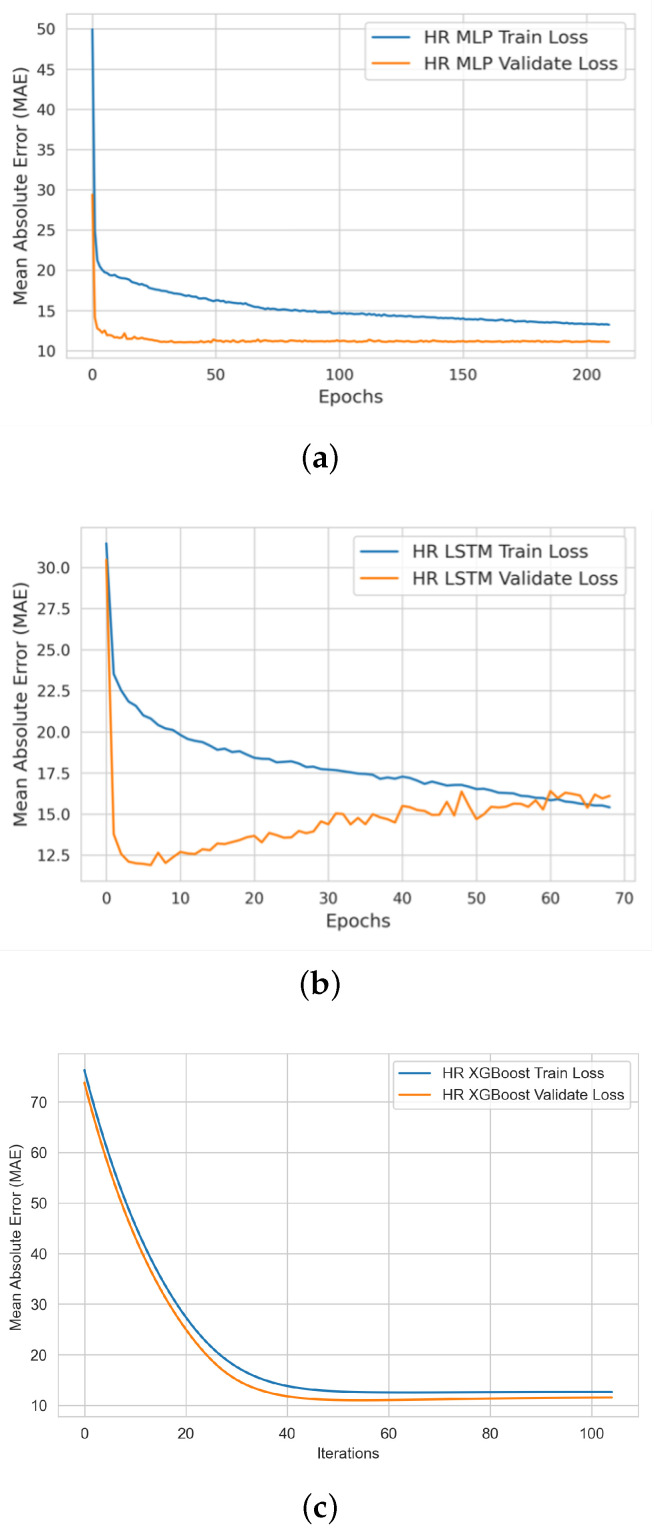
Heart rate ML models’ performance curves during the learning and validation of the (**a**) MLP, (**b**) LSTM, and (**c**) XGBoost models.

Figure 6 shows the profiles of the MLP, LSTM, and XGBoost ML models during the HR model training and validation experiments. Our analysis showed that the HR XGBoost model provided the best learning and validation performance in comparison with the MLP and LSTM models.

#### 4.2.2. BR ML Model Training and Validation

**Figure 7:**
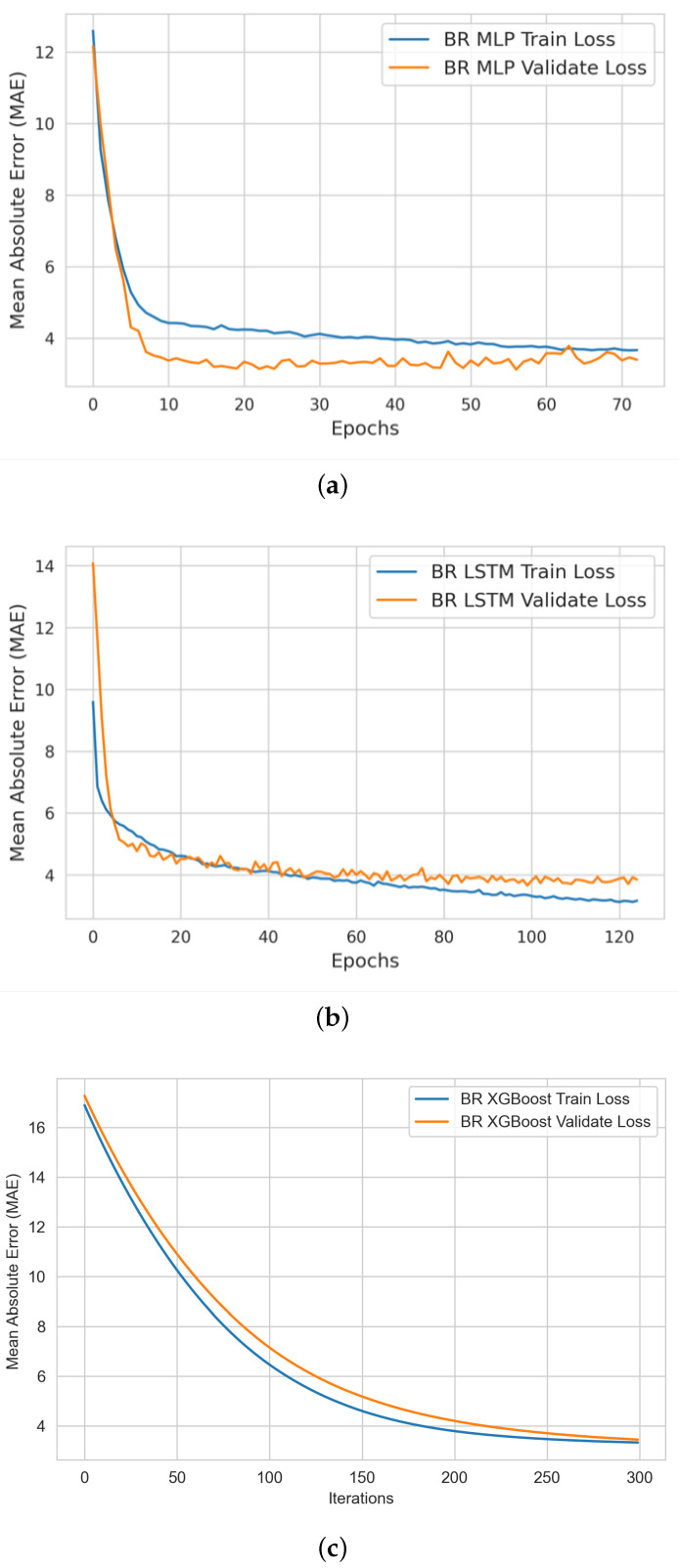
Breathing rate ML models’ performance curves during learning and validation of (**a**) MLP (**b**) LSTM and (**c**) XGBoost models.

Figure 7 shows the profile of the MLP, LSTM, and XGBoost ML models during the BR model training and validation experiments. Our analysis showed that the BR XGBoost model provided the best learning and validation performance in comparison with the MLP and LSTM models.

#### 4.2.3. SpO2 ML Model Training and Validation

**Figure 8:**
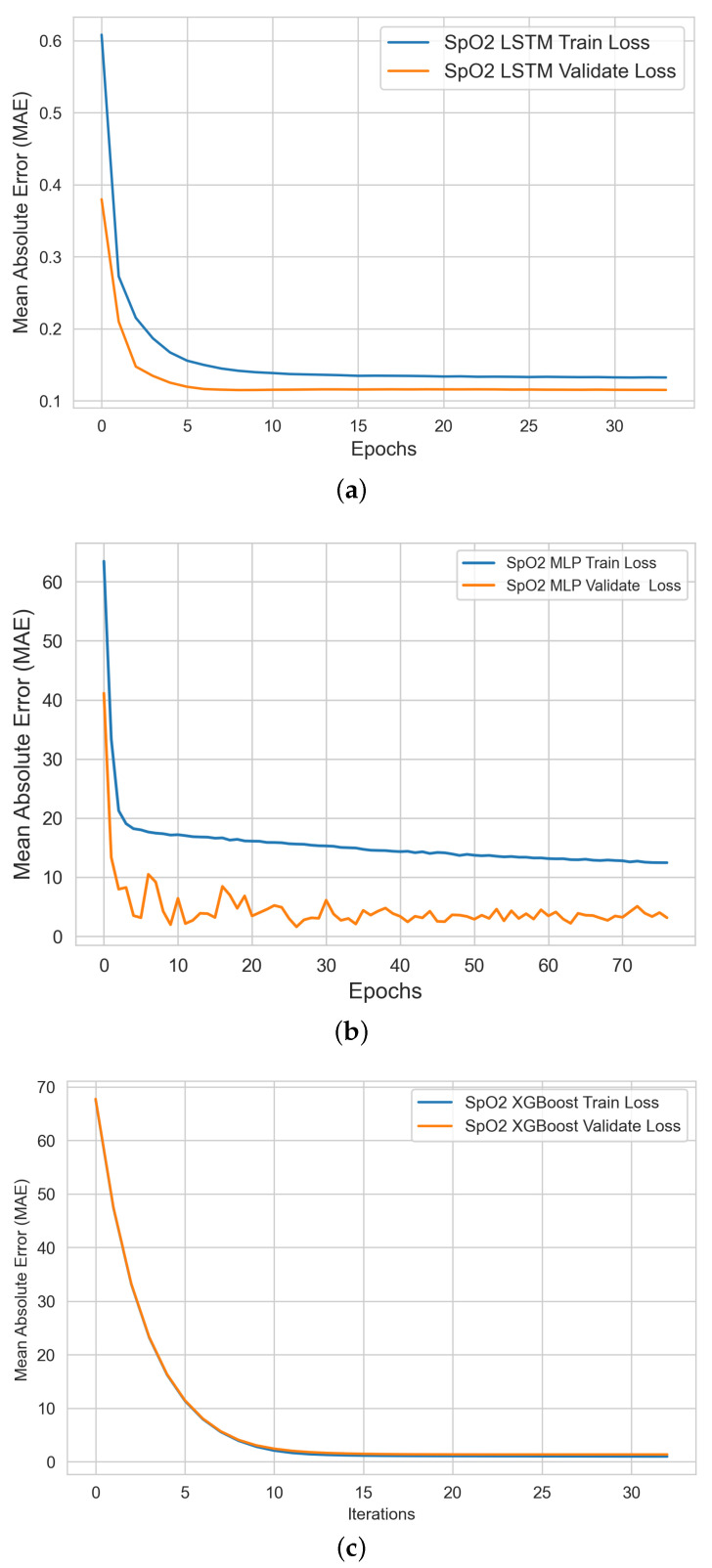
SpO2 level ML models’ performance curves during the learning and validation of the (**a**) MLP, (**b**) LSTM, and (**c**) XGBoost models.

Figure 8 shows the profile of MLP, LSTM, and XGBoost ML models during SpO2 model training and validation experiments. Our analysis shows that the SpO2 XGBoost model provides the best learning and validation performance in comparison to MLP and LSTM models.

### 4.3. Testing Results

#### 4.3.1. HR ML Model Testing

Table 4 shows the performance of the tested HR ML models. Our analysis showed that the XGBoost model provided the lowest testing performance error rate (9.5%) in comparison with the MLP and LSTM models. XGBoost showed excellent performance in terms of high correlation (0.73) between the ground-truth signal and the estimated output signal from the HR XGBoost model than the estimated HR signals obtained from the MLP and LSTM models.

#### 4.3.2. BR ML Model Testing

Table 5 demonstrates the performance of the tested BR ML models. Our analysis showed that the XGBoost model provided the lowest testing performance error rate (13.4%) in comparison with the MLP and LSTM models. XGBoost showed a higher correlation (0.59) between the ground-truth signal and the estimated output signal from the BR XGBoost model than the estimated BR signals obtained from MLP and LSTM models

#### 4.3.3. SpO2 ML Model Testing

Table 6 highlights the performance of the tested SpO2 ML models. Our analysis showed that the XGBoost model provided the best performance (lower testing performance error rate (1.1%) and a higher correlation (0.54) between the ground-truth signal and the estimated output signal from the BR XGBoost model) than the MLP and LSTM models

## 5. Discussion

### 5.1. System Runtime

**Figure 9:**
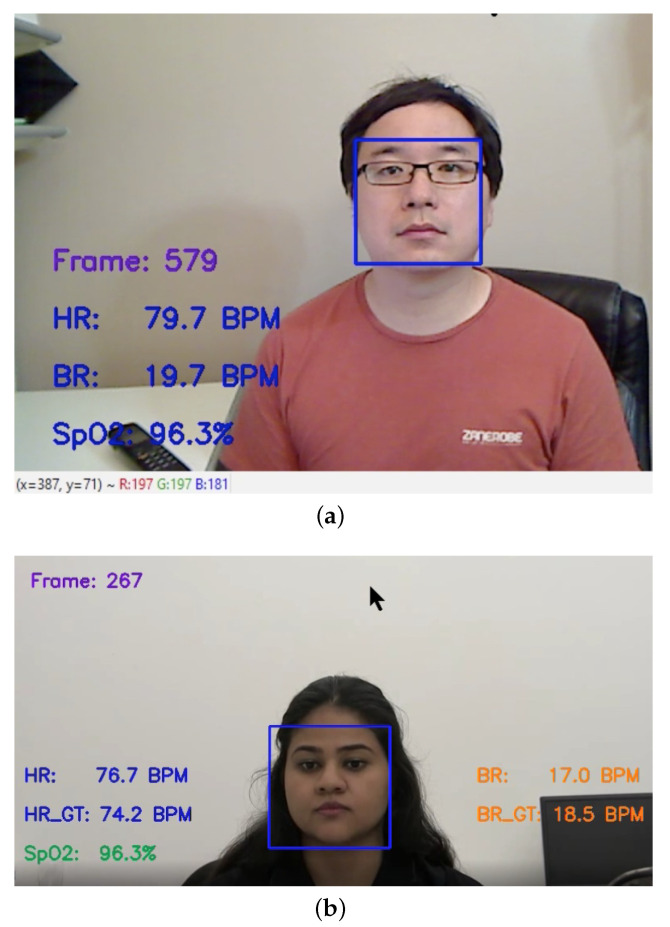
Demonstration of the ML-based multi-bio-signal DT model results: (**a**) real-time demo, (**b**) offline demo.

The ML-based bio-signal DT model’s output interface is shown in Figure 9. The real-time window updates the bio-signal readings every 30 frames (i.e., every second if the camera recording speed is 30 FPS). The system takes 40–60 milliseconds to process all stages of the bio-signal data extraction module and takes 64–66 milliseconds to measure all bio-signals in real time. This computational measurement is based on the hardware specification stated in Appendix A. Figure 9a shows the system’s output interface using a video camera as a real-time input source, where HR denotes the heart rate, BR denotes the breathing rate, and SpO2 denotes the blood oxygen level.

Figure 9b shows the system’s output interface based on an offline video input source. The remotely measured HR, BR, and SpO2 are published on the screen. The system reads and updates the ground-truth values for the heart rate and breathing rate (HR GT and BR GT), respectively, from pre-synchronized data files for easy comparison purposes.

The DT system showed great performance in modelling and measuring the HR, BR, and SpO2 bio-signals because the testing environment conditions were identical or close to those of the lab environment during the dataset collections. This is because machine learning theory only deals with generalizations within the same distribution between the training and validation/testing datasets, i.e., independent and identically distributed (i.i.d.) assumptions.

Two measures were considered during the DT system design to manage system sensitivity to noise: (i) we used the green-channel data in the RGB colour space due to its robustness to bio-signal extraction (the presence of the highest signal-to-noise ratio (SNR) on the bio-signal data was in this channel); (ii) we used three regions of interest (ROI) on the face frame (left and right cheeks and the forehead) because the signals related to the absorption bands for oxy- and de-oxy hemoglobin had the strongest SNR corresponding to blood volume changes in these facial skin regions.

The merits of this initial system are that it (1) demonstrates a simple DT architecture that uses a shared common (bio-signal data extraction) module that takes a single input (raw video frames), processes it using a light-weight pre-trained ML model for each bio-signal and produces multiple outputs (three estimated outputs: heart rate, respiratory rate, and blood oxygen saturation level); (2) demonstrates promising modelling and fusion performance; (3) engineers light-weighted ML DT models capable of working in both real-time and offline modes and enables rapid deployment in the production environment due to its fast processing time and efficient hardware requirements, which establishes our pioneering research work as a new milestone for adapting ML-based DT technology solutions to real-time metaverse digital health applications; and (4) enables potential digital health data security through computer vision and biometrics with the data fusion of multiple bio-signals.

Our XGBoost model showed consistently outstanding performance in estimating all three bio-signals. The advantages of XGBoost over the MLP and LSTM models are that (1) it has immunity and robustness against anomalies and outliers; this is because it is a tree-based ensemble learning technique that uses the boosting method to build each tree on previous tree residuals/errors. Outliers will have much larger residuals than non-outliers, will not be considered during tree splitting, and will not contribute to the overall model error value during training; (2) it is built on a simple architecture with a small number of parameters for optimization; (3) it can be configured, trained, and tested quickly; and (4) it does not require input data normalization; (5) it generates a small, trained model size, which is light enough (less than 700 KB) to enable rapid deployment in future production environments.

The XGBoost model performance was optimized after tuning three hyperparameters (learning rate, no. of estimators (number of trees), and maximum depth (depth of trees)). In addition, we fixed the random state value to enable reproducible results. We applied the early stopping technique to prevent the model from overfitting the training data (e.g., stopping the model training when the validation loss stopped improving after 50 iterations for the HR model). The full design parameters of the best-performing XGBoost regression models used in our DT system are shown in Table 7.

### 5.2. Limitations and Future Work

The limitations we experienced during this work were as follows: (a) Since most of the heart rate data we collected were within the 65–85 BPM spectrum, heart rate data beyond this spectrum are highly desired for further model refinement, particularly for detecting heart rates at an exercising level; (b) Some datasets, for example, the COHFACE dataset, came with highly compressed video files and the original recordings were noisy. These could not contribute to the model training because weak signals representing blood volume changes were already filtered during the video compression process; (c) The hardware variations, such as different webcam specifications and real-time light conditions, can all contribute to reducing accuracy in the performance; and (d) We noticed that the COHFACE dataset provided raw blood volume pulse (BVP) and breathing recordings and we needed to convert them into HR and BR data in the frequency domain using BioSPPy (Python bio-signal processing library) before we could use them as ground truths. However, this introduced a slight delay in the video physical data synchronization as every processed HR/BR data required a set of raw inputs before making calculations.

Although light and vibration/motion are factors that are involved in ML models’ robustness, our research focused on the accuracy of the DT system itself. Studying the impact of such factors, including quality index analysis, is a potential area of future research.

Therefore, to tackle these challenges, our future work will focus on (i) using generative models to enrich each bio-signal dataset with more data and features such as age, gender, and skin colour; (ii) adding a motion-based feature to improve the accuracy of the BR model; and (iii) developing advanced hybrid ML ensemble model architectures with transfer learning (e.g., autoencoder with MLP or XGBoost).

## 6. Conclusions

The contribution of this research paper focused on addressing an important gap in the literature. This gap involved the need to demonstrate how to develop and implement DT technology concept for modern human health and well-being applications in the metaverse at a granular level. To the best of the authors’ knowledge, no previous work has demonstrated a fully functional human DT prototype that integrates machine learning and computer vision/rPPG technologies, capable of detecting tiny colour-intensity changes directly from human facial video frames without making physical contact, and then fusing three human physiological bio-signals in real time concurrently (heart rate, breathing rate, and blood oxygen saturation level) using simple ML regression models with minimum pre-processing/processing time and very efficient memory hardware requirements.

Understanding the concept of the DT and how it is implemented in the human DT domain is a challenging research area. To address this challenge, we presented the novelty of our work simply and comprehensively. The engineered solution was experimented with and validated under both real-time and offline configurations. The system performances under our testing achieved strong accuracy in the modeling and measuring of HR, BR, and SpO2 bio-signals. The HR ML model’s testing results showed that the XGBoost model provided the lowest testing performance error rate (9.5%) in comparison with the MLP and LSTM models, which meant a 91% accuracy in modeling and measuring the HR bio-signal; the BR XGBoost model provided the lowest testing performance error rate (13.4%, i.e., 87% accuracy); and the SpO2 XGBoost model provided the lowest testing performance error rate (1.1%, i.e., 99% accuracy).

We consider our pioneering research work a new milestone in establishing the foundation and path forward for realizing a holistic human health and well-being DT model for real-world personalized health applications and virtual telemedicine services.

## Figures and Tables

**Table 1:** Statistical Summary of the HR Engineered Datasets Based on the Extracted Green-Channel Data.

Dataset	Count	Mean	STD	Min	25%	50%	75%	Max
VIPL	36,291	80.8	14.8	47.0	72.0	79.0	87.5	255
Our Lab	18,425	78.1	23.7	52.2	63.6	70.4	78.4	165
COHFACE	4180	74.0	14.8	48.7	62.6	71.2	84.1	153

**Table 2:** Statistical Summary of the BR Engineered Datasets Based on the Extracted Green-Channel Data.

Dataset	Count	Mean	STD	Min	25%	50%	75%	Max
Our Lab	18,425	18.0	5.5	3.0	14.7	17.4	19.9	55
COHFACE	4180	713.2	3.4	5.1	10.8	12.7	15.9	21

**Table 3:** Statistical Summary of the SpO2 Engineered Datasets Based on the Extracted Green-Channel Data.

Dataset	Count	Mean	STD	Min	25%	50%	75%	Max
VIPL	36,291	96.6	5.6	43.7	96.0	97.0	98.0	127

**Table 4:** Testing Performance Results of the HR ML Models.

HR ML Model	MAE	RMSE	MAPE %	Pearson Correlation
MLP	7.5	9.5	12.0	0.66
LSTM	114.1	17.6	19.6	0.55
XGBoost	7.0	8.8	9.5	0.73

**Table 5:** Testing Performance Results of the BR ML Models.

BR ML Model	MAE	RMSE	MAPE %	Pearson Correlation
MLP	2.6	3.2	13.9	0.42
LSTM	3.3	3.8	17.5	0.09
XGBoost	2.5	2.9	13.4	0.59

**Table 6:** Testing Performance Results of the SpO2 ML Models.

BR ML Model	MAE	RMSE	MAPE %	Pearson Correlation
MLP	2.9	3.2	3	0.01
LSTM	1	1.3	1.1	0.06
XGBoost	1	1.4	1.1	0.54

**Table 7:** Design parameters of the best-performing XGBoost regression models.

Parameter	HR Model	BR Model	SpO2 Model
Learning rate	0.05	0.05	0.01
No. of estimators	350	300	100
Maximum depth	4	5	6
Random state	99	99	99
Early stopping rounds	50	200	5
Evaluation metric	MAE	MAE	MAE

## Data Availability

Not applicable.

## Abbreviations

The following abbreviations are used in this manuscript:

HR	Heart Rate
BR	Breathing Rate
DT	Digital Twin
MLP	Multilayer Perceptron
EDA	Exploratory Data Analysis
LSTM	Long Short-Term Memory
RNN	Recurrent Neural Networks
XGBoost	eXtreme Gradient Boosting
FPS	Frames Per Second
DL	Deep Learning
ReLU	Rectified Linear Unit

## Appendix A. DT System Hardware and Software Configuration

Our DT model implementation is compatible with laptop and desktop computer configurations equipped with regular webcam hardware. A fast CPU and a large RAM size are preferred for speeding up the camera frame rate. For an ideal bio-signal measurement, the user should have clean facial skin exposed without makeup or sunscreen, and the light on the face should be equally bright.

The user needs to breathe normally and maintain a stable sitting-still or standing pose in front of the camera and within 0.5 to 1.5 m.

We implemented our system and research experimentation using Python (ver. 3.5), Microsoft Windows 10 (ver. 2004), PyCharm IDE (ver. 2020.1.2), and Anaconda3 (ver. 4.8.3). The hardware specifications of our system and research experimentation are based on two configurations: (i) Asus Laptop (Intel Core i5 8440U 1.7Ghz CPU, NVIDIA MX150 GPU, 16GB RAM, 512GB SSD Hard drive), and (ii) Desktop PC (Intel Core i7 8700K 3.7Ghz CPU, NVIDIA Geforce 1070 GPU, 32GB RAM, 1TB SSD Hard drive).

## References

[1] El Saddik A., Laamarti F., Alja’Afreh M. (2021). The Potential of Digital Twins. IEEE Instrum. Meas. Mag..

[2] Gámez Díaz R., Yu Q., Ding Y., Laamarti F., El Saddik A. (2020). Digital Twin Coaching for Physical Activities: A Survey. Sensors.

[3] Sahal R., Alsamhi S.H., Brown K.N. (2022). Personal Digital Twin: A Close Look into the Present and a Step towards the Future of Personalised Healthcare Industry. Sensors.

[4] Costantini A., Di Modica G., Ahouangonou J.C., Duma D.C., Martelli B., Galletti M., Antonacci M., Nehls D., Bellavista P., Delamarre C. (2022). IoTwins: Toward Implementation of Distributed Digital Twins in Industry 4.0 Settings. Computers.

[5] Segovia M., Garcia-Alfaro J. (2022). Design, Modeling and Implementation of Digital Twins. Sensors.

[6] da Silva Mendonça R., de Oliveira Lins S., de Bessa I.V., de Carvalho Ayres F.A., de Medeiros R.L.P., de Lucena V.F. (2022). Digital Twin Applications: A Survey of Recent Advances and Challenges. Processes.

[7] El Saddik A. (2018). Digital twins: The convergence of multimedia technologies. IEEE Multimed..

[8] Laamarti F., Badawi H.F., Ding Y., Arafsha F., Hafidh B., Saddik A.E. (2020). An ISO/IEEE 11073 Standardized Digital Twin Framework for Health and Well-Being in Smart Cities. IEEE Access.

[9] Muhammad G., Alshehri F., Karray F., Saddik A.E., Alsulaiman M., Falk T.H. (2021). A comprehensive survey on multimodal medical signals fusion for smart healthcare systems. Inf. Fusion.

[10] Cheng C.H., Wong K.L., Chin J.W., Chan T.T., So R.H.Y. (2021). Deep Learning Methods for Remote Heart Rate Measurement: A Review and Future Research Agenda. Sensors.

[11] Ma X., Tobón D.P., El Saddik A., McDaniel T., Berretti S., Curcio I.D.D., Basu A. (2020). Remote Photoplethysmography (rPPG) for Contactless Heart Rate Monitoring Using a Single Monochrome and Color Camera. Proceedings of the Smart Multimedia.

[12] Niu X., Han H., Shan S., Chen X. (2018). VIPL-HR: A multi-modal database for pulse estimation from less-constrained face video. Proceedings of the Asian Conference on Computer Vision.

[13] Wang H., Zhou Y., Saddik A.E. (2021). VitaSi: A real-time contactless vital signs estimation system. Comput. Electr. Eng..

[14] Atienza R. (2020). Advanced Deep Learning with TensorFlow 2 and Keras: Apply DL, GANs, VAEs, Deep RL, Unsupervised Learning, Object Detection and Segmentation, and More.

[15] Ioffe S., Szegedy C. Batch Normalization: Accelerating Deep Network Training by Reducing Internal Covariate Shift. Proceedings of the 32nd International Conference on International Conference on Machine Learning.

[16] Goodfellow I., Bengio Y., Courville A. (2016). Deep Learning (Adaptive Computation And Machine Learning Series).

[17] Kingma D.P., Ba J. (2014). Adam: A method for stochastic optimization. arXiv.

[18] Hochreiter S., Schmidhuber J. (1997). Long short-term memory. Neural Comput..

[19] Chen T., Guestrin C. Xgboost: A scalable tree boosting system. Proceedings of the 22nd ACM SIGKDD International Conference on Knowledge Discovery and Data Mining.

[20] Chen D.Y., Wang J.J., Lin K.Y., Chang H.H., Wu H.K., Chen Y.S., Lee S.Y. (2014). Image sensor-based heart rate evaluation from face reflectance using Hilbert–Huang transform. IEEE Sens. J..

[21] Wang C., Pun T., Chanel G. (2018). A comparative survey of methods for remote heart rate detection from frontal face videos. Front. Bioeng. Biotechnol..

[22] Lévesque L. (2014). Nyquist sampling theorem: Understanding the illusion of a spinning wheel captured with a video camera. Phys. Educ..

[23] Johns Hopkins Medicine Health Library: Vital Signs (Body Temperature, Pulse Rate, Respiration Rate, Blood Pressure). https://www.hopkinsmedicine.org/health/conditions-and-diseases/vital-signs-body-temperature-pulse-rate-respiration-rate-blood-pressure.

